# Exploring the Impact of Vitamin D and Zinc Deficiencies on Seborrheic Dermatitis: A Comparative Study

**DOI:** 10.1002/hsr2.70283

**Published:** 2024-12-24

**Authors:** Amine Kashiri, Negin Maghsoudloo

**Affiliations:** ^1^ Gorgan Faculty of Medicine Golestan University of Medical Sciences Gorgan Iran

**Keywords:** case‐control study, disease severity, seborrheic dermatitis, vitamin D deficiency, zinc levels

## Abstract

**Background and Aims:**

Seborrheic dermatitis (SD) is a chronic inflammatory skin condition that affects patients' quality of life. Emerging evidence suggests that vitamin and mineral deficiencies may contribute to its progression, although the exact etiology remains unclear.

**Objective:**

This case‐control study assessed the serum levels of vitamin D and zinc in SD patients compared to a healthy control group, with a focus on how these deficiencies relate to disease severity.

**Methods:**

A total of 71 SD patients and 71 healthy controls participated, providing demographic data and blood samples. Disease severity was evaluated using the SEDASI scoring system. Serum vitamin D and zinc levels were measured using ELISA and photometric methods, respectively. Statistical analysis was performed using t‐tests and chi‐square tests in SPSS 24, with groups matched for demographic variables (*p* > 0.05).

**Results:**

Findings revealed no significant difference in vitamin D levels between SD patients and the control group (*p* = 0.066). However, zinc levels were notably lower in the SD group (*p* = 0.001). Additionally, vitamin D levels were inversely related to the severity of SD (*p* = 0.022), while no correlation was found between zinc levels and disease severity (*p* = 0.664).

**Conclusion:**

Zinc deficiency appears to play a role in SD pathogenesis, while lower vitamin D levels are associated with increased disease severity. These findings highlight the need for further research into the potential therapeutic role of nutrient supplementation in managing SD.

## 1 Introduction

Seborrheic dermatitis (SD) is a chronic and recurrent inflammatory skin condition that majorly impacts areas with a high concentration of sebaceous glands, such as the scalp, face, and chest. It is characterized by symptoms like erythema, scaling, and itching, significantly affecting individuals' quality of life and psychological well‐being. Despite its prevalence, the exact cause of SD remains unclear, with evidence suggesting that genetic, environmental, and immunological factors contribute to its development. The yeast *Malassezia* plays a central role in exacerbating SD symptoms, further complicating its pathogenesis. SD affects individuals of all ages but is more common in men and certain age groups. Its presentation can range from mild dandruff to severe redness and flaking. Factors such as hormonal changes, immune status, environmental conditions, and diet have been implicated in its development, with the role of *Malassezia* particularly significant in triggering the inflammatory response [1, 2].

Recent studies have suggested a link between deficiencies in certain micronutrients, notably vitamin D and zinc, and the onset or severity of skin conditions, including SD. Vitamin D is known for its immunomodulatory and anti‐inflammatory properties, which are vital for skin health and immune function. Similarly, zinc is essential for numerous biological processes and supports skin integrity and immune responses. However, the relationship between these nutrient deficiencies and SD remains unclear, with inconsistent findings in the literature [3, 4].

Recent studies have explored the relationship between SD and serum levels of specific nutrients, revealing contradictory results that highlight the complexity of this skin condition. Akbaş et al. [3] discovered a significant association between severe vitamin D deficiency and SD, with a higher prevalence of deficiency observed in younger patients and women. Similarly, Karabay and Çerman [5] identified significantly lower serum zinc levels in patients with SD compared to healthy controls, suggesting that deficiencies in vitamin D and zinc might play roles in the development or exacerbation of SD. However, other studies, such as those by Hajheydari, Saeedi, and Hosseinzadeh [6] and Jahan et al. [7], did not find significant differences in serum levels of selenium, zinc, copper, and other trace elements and nutrients between SD patients and healthy individuals, indicating that the influence of these nutrients on SD may vary or require further investigation to fully understand [6, 7, 8].

The diverse findings across these studies underscore the need for continued research into the nutritional and immunological aspects of SD. While some research points to a potential link between nutrient deficiencies and SD, other studies challenge this notion, suggesting that the relationship between SD and serum levels of certain nutrients, such as vitamin D, zinc, selenium, and copper, may not be straightforward. These discrepancies highlight the importance of considering a broad range of factors when studying SD, including genetic predispositions, environmental triggers, and individual health statuses. Ultimately, a more nuanced understanding of how these elements interact could lead to more effective prevention and treatment strategies for SD, improving patient outcomes and quality of life [9, 10]. This study aims to clarify the relationship between serum levels of vitamin D and zinc in individuals with SD compared to a healthy control group, focusing on whether deficiencies in these nutrients contribute to SD pathogenesis or exacerbate the condition.

## 2 Materials and Methods

This study received approval from the local ethics committee, and all participants provided written informed consent. It was designed as a cross‐sectional case‐control study with a descriptive and analytical approach, targeting patients with SD at the Deziani clinic in Gorgan City, northern Iran.

### 2.1 Inclusion and Exclusion Criteria

Participants were included if they had a confirmed diagnosis of SD by a physician, provided consent, could communicate effectively, and had no psychiatric illness. Individuals were excluded if they had skin conditions associated with vitamin D deficiency (e.g., atopic dermatitis, psoriasis, SLE), used vitamin D or zinc supplements in the past 6 months, or had a history of medications affecting vitamin D metabolism, such as colchicine or certain anticonvulsants.

### 2.2 Sample Size Calculation

The sample size was calculated based on the study by Rahimi, Nemati, and Shafaei‐Tonekaboni [11] and by using the formula below, with a confidence level of 0.95 and a test power of 0.8, considering a one‐sided alternative hypothesis using the means and standard deviations of vitamin D in both the SD patient group and the control group, resulting in 78 individuals per group. Additionally, the sample size considering the mean values of Zn in both the SD patient group and the control group, derived from the results of the study by Karabay and Çerman [5], with a confidence level of 0.95 and a test power of 0.8, and considering a one‐sided alternative hypothesis, was determined to be 64 individuals per group according to the formula below:

$$n_{1}=n_{2}=\left[\frac{{\left(Z_{1-\alpha }+Z_{1-\beta }\right)}^{2}\left[{\mathrm{SD}}_{1}^{2}+{\mathrm{SD}}_{2}^{2}\right]}{{\left(\mu_{2}-\mu_{1}\right)}^{2}}\right].$$

The final sample size was calculated based on the average of the two studies to be 71 individuals for each group. Sampling was done on an accessible basis from all individuals eligible to participate in the study after obtaining informed consent.

### 2.3 Study Groups

The study included two groups: Group 1 (patients with SD) and Group 2 (healthy controls), selected from patients' companions. After obtaining permission and consent, participants were informed about the study's objectives, and demographic data, including age, were collected via a checklist. Both groups underwent general and dermatological examinations, with disease severity classified as mild, moderate, or severe by a specialist.

### 2.4 Biochemical Analysis

For serum vitamin D and zinc levels, 2 cc of fasting venous blood was drawn under sterile conditions. Measurements were performed at the Deziani Hospital laboratory. Vitamin D levels were assessed using an ELISA kit and categorized as deficiency (< 20 ng/dL), insufficient (21–29 ng/dL), and sufficient (> 30 ng/dL). Zinc levels were measured using a photometric kit and an autoanalyzer, with levels < 70 µg/dL considered deficient.

### 2.5 Study Design

This study was a case‐control design that included 142 individuals, with 71 patients diagnosed with SD and 71 healthy controls. Patient inclusion criteria were prespecified, and the control group was matched for age, gender, and key demographic factors. The study followed the Strengthening the Reporting of Observational Studies in Epidemiology (STROBE) guidelines, and statistical analyses were conducted as described below.

### 2.6 Statistical Analysis

All statistical analyses were performed using SPSS software (IBM SPSS Statistics V. 29), with a significance level (alpha) set at *p* < 0.05. All tests were two‐sided unless otherwise specified. Means and standard deviations were calculated for continuous variables that were normally distributed, while medians and interquartile ranges (IQR) were used for non‐normally distributed data. Categorical variables were expressed as frequencies and percentages. The actual numerators and denominators for percentages were provided where relevant. Between‐group comparisons for continuous variables were conducted using independent *t*‐tests (for normally distributed data) or the Mann–Whitney *U*‐test (for non‐normally distributed data). Chi‐square tests were used to compare categorical variables between the patient and control groups.

Subgroup analyses were conducted to explore relationships between disease severity and nutrient levels (e.g., vitamin D and zinc) among patients. These analyses were exploratory and were not prespecified in the study protocol. The primary outcomes of interest—serum levels of 25(OH) vitamin D and zinc—were prespecified. Comparisons between these biomarkers and demographic factors such as age, gender, and disease severity were analyzed with a focus on identifying statistically significant differences. *p* Values were calculated for all comparisons, with the following conventions: *p* < 0.001, *p* values between 0.001 and 0.01 were reported to the nearest thousandth, and those greater than or equal to 0.01 were reported to the nearest hundredth. Where possible, findings were presented with 95% confidence intervals (CIs) to provide a measure of the precision of the estimates. Data were analyzed using ANOVA or Kruskal–Wallis test depending on the normality of the distribution.

### 2.7 Disease Severity Assessment

The severity of SD was evaluated using the SEDASI (Seborrheic Dermatitis Area and Severity Index). The SEDASI score assesses both the area of involvement and the severity of erythema, scaling, and pruritus on a scale of 0–3. The total score categorizes patients as having mild (0–3), moderate (4–6), or severe (7+) disease severity. The area of involvement was calculated as a percentage of affected regions, while erythema, scaling, and pruritus were graded for severity [12].

### 2.8 Ethical Considerations

In this research, all ethical guidelines were observed in accordance with the National Ethics Guidelines for Publication of Medical Science Research Works (2008–2009). To this end:

- The proposed plan was implemented after approval by the University Research Council and the Ethics Committee of Golestan University of Medical Sciences.
- The information of patients participating in the research was kept confidential. For this purpose, numerical codes without using names and surnames were used for coding their information, and confidentiality was maintained at all stages of the research.
- Written consent was obtained from all participants in this study, and full explanations about the study's objectives were provided. The name of the author(s) of the plan was fully disclosed, and they accepted responsibility for the content of the plan.
- Ethics approval was obtained from the Golestan University of Medical Sciences, Research Ethics Committee (Gorgan, Iran).

## 3 Results

In this study, 142 individuals were included, comprising 71 patients (50.0%) and 71 control subjects (50.0%). The mean age of the patients was 31.5 years (standard deviation = 13.2), while the mean age of the control group was 35.5 years (standard deviation = 13.5). No significant difference in age was observed between the two groups (*p* = 0.580). The demographic characteristics of both groups are presented in Table 1. There were no significant differences in gender, ethnicity, residence, education, and occupation, indicating homogeneity (*p* > 0.05).

**Table 1:** Comparison of demographic characteristics of the two study groups.

Factor	Frequency (%)		*p* value
	Patient	Control	
Gender			
Woman	45 (63.4)	46 (64.8)	0.861
Man	26 (36.6)	25 (35.2)	
Ethnicity			
Persian	59 (82.5)	50 (70.5)	0.418
Turkmen	2 (3.2)	4 (5.6)	
Sistani	7 (9.5)	13 (18.3)	
Other	3 (4.8)	4 (5.6)	
Residence			
City/urban	57 (80.0)	54 (76.1)	0.654
Village/rural	14 (20.0)	17 (23.9)	
Education			
Illiterate	3 (4.2)	3 (4.2)	0.632
Diploma and below	34 (47.9)	39 (54.9)	
University level	34 (47.9)	29 (40.9)	
Occupation			
Employee	12 (16.9)	11 (15.5)	0.762
Housewife	20 (28.2)	24 (33.8)	
Self‐employed	9 (12.7)	11 (15.5)	
Other	30 (42.2)	25 (35.2)	

**Figure 1:**
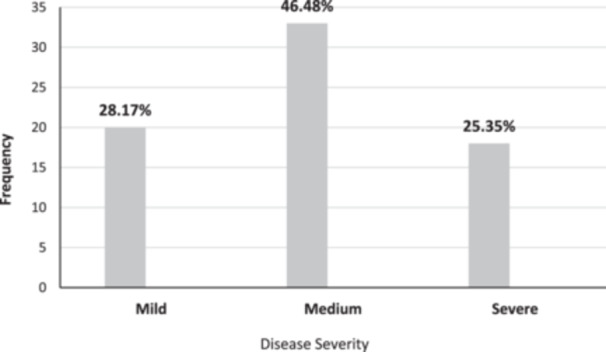
Frequency distribution of disease severity in the patient group. Mild (*n* = 20). Medium (*n* = 33). Severe (*n* = 18).

Figure 1 illustrates the distribution of SD severity among the patient group as diagnosed by a specialist physician. Nearly half of the patients experienced the disease with moderate severity, accounting for 46.48% of the patient group.

The comparison of serum levels of 25(OH) vitamin D and zinc (Zn) between the patient and control groups are presented in Table 2. No significant difference was found in the serum levels of 25(OH) vitamin D between the groups (mean ± standard deviation: patients 18.3 ± 9.2 nmol/L; controls 20.5 ± 8.7 nmol/L; *p* = 0.066). However, the serum zinc levels in the patient group were significantly lower than those in the control group (mean ± standard deviation: patients 10.5 ± 3.1 μmol/L; controls 14.7 ± 3.6 μmol/L; *p* < 0.001). This suggests that zinc deficiency is more prevalent among patients with SD compared to healthy controls, indicating a potential role of zinc in the pathology or management of the disease.

**Table 2:** Comparison of serum levels of 25(OH) vitamin D and zinc between patient and control groups.

Factor	Mean		*p* value
	Patient	Control	
25(OH) vit D (ng/dL)	34.5 (19.0)	37.1 (12.6)	0.066
Zn (µg/dL)	68.9 (16.8)	76.7 (17.4)	0.001

**Figure 2:**
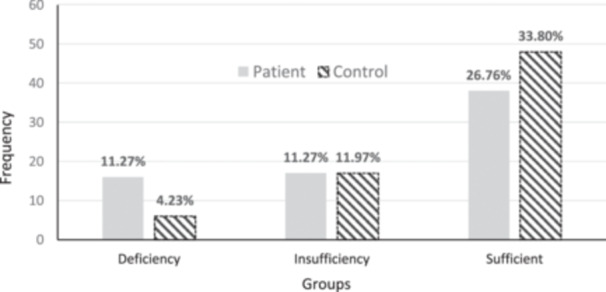
Comparison of vitamin D sufficiency between patient and control groups. Patients: Deficiency (*n* = 16), Insufficiency (*n* = 17), Sufficient (*n* = 38). Control: Deficiency (*n* = 6), Insufficiency (*n* = 17), Sufficient (*n* = 48).

**Figure 3:**
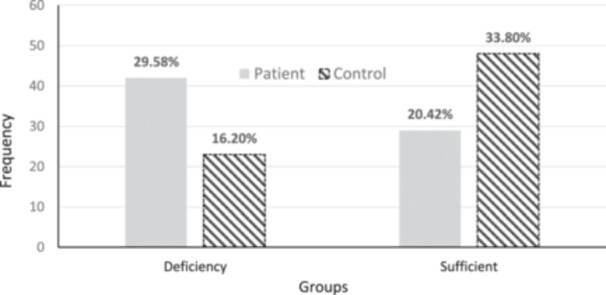
Comparison of zinc sufficiency between the patient and control groups.

Although the prevalence of vitamin D deficiency was higher in the patient group compared to the control group, the comparison of vitamin D sufficiency levels did not show a statistically significant difference (*p* = 0.058; Figure 2). This suggests a trend towards lower vitamin D levels among patients, but the difference does not reach statistical significance, implying that other factors may contribute or that a larger sample size might be needed to detect a significant difference. The comparison of zinc sufficiency levels between the groups showed a significantly higher prevalence of individuals with zinc deficiency in the patient group (*p* < 0.001; Figure 3).

Analysis of vitamin D levels in the patient group indicated that the average vitamin D concentration was significantly lower in patients with moderate and severe forms of SD compared to those with mild cases (mean ± standard deviation: moderate 16.8 ± 8.1 nmol/L, severe 15.2 ± 7.5 nmol/L; mild 22.7 ± 9.8 nmol/L; *p* = 0.022). In contrast, Zn levels did not exhibit significant variation across different severities of the disease (*p* = 0.664; see Table 3).

**Table 3:** Comparison of serum levels of 25(OH) vitamin D and zinc in the patient group based on disease severity.

Variable	Mild	Intermediate	Severe	*p* value
25(OH) Vitamin D (ng/dL)	46.0 (23.8)	30.3 (14.1)	30.0 (16.9)	0.022
Zn (µg/dL)	71.6 (18.3)	69.3 (16.3)	65.0 (16.0)	0.664

Table 4 presents the comparison of vitamin D sufficiency levels based on disease severity, revealing no significant differences in the frequency of sufficiency among patients with varying disease severity. Similarly, Table 5 shows the comparison of zinc sufficiency levels, where no significant differences were found across different severity levels.

**Table 4:** Comparison of 25(OH) vitamin D sufficiency based on disease severity.

Variable	Mild (%)	Intermediate (%)	Severe (%)	*p* value
Sufficient (≥ 30 ng/mL)	15 (39.5)	15 (39.5)	8 (21.0)	0.166
Insufficient (21–29 ng/mL)	4 (23.5)	8 (47.1)	5 (29.4)	
Deficient (≤ 20 ng/mL)	1 (6.3)	10 (62.5)	5 (31.2)	

**Table 5:** Comparison of zinc adequacy based on disease severity.

Variable	Mild (%)	Intermediate (%)	Severe (%)	*p* value
Sufficient (> 70 ng/mL)	7 (24.1)	15 (51.8)	7 (24.1)	0.166
Deficient (≤ 70 ng/mL)	13 (30.9)	18 (42.9)	11 (26.2)	
Sufficient (> 70 ng/mL)	7 (24.1)	15 (51.8)	7 (24.1)	

When examining vitamin D sufficiency based on gender, as shown in Table 6, no significant differences were observed in the frequency of vitamin D levels among male and female patients (*p* = 0.320). Table 7 presents the comparison of zinc sufficiency levels by gender, which also showed no significant differences in frequency (*p* = 0.420).

**Table 6:** Comparison of 25(OH) vitamin D sufficiency in the patient group based on gender.

Variable	Female (%)	Male (%)	*p* value
Sufficient (≥ 30 ng/mL)	24 (63.2)	14 (36.8)	0.421
Insufficient (21–29 ng/mL)	9 (52.9)	8 (47.1)	
Deficient (≤ 20 ng/mL)	12 (75.0)	4 (25.0)	

**Table 7:** Comparison of zinc sufficiency in the patient group based on gender.

Variable	Female (%)	Male (%)	*p* value
Sufficient (< 70 ng/mL)	16 (55.2)	13 (44.8)	0.233
Deficient (≥ 70 ng/mL)	29 (69.0)	13 (31.0)	

These findings suggest that while the severity of SD impacts the average level of vitamin D, with lower levels observed in more severe cases, the severity does not similarly affect zinc levels. Additionally, neither vitamin D nor zinc levels showed significant differences when analyzed based on gender, indicating that these nutrient levels are not influenced by gender among patients with SD.

## 4 Discussion

This comparative study investigated the serum levels of vitamin D and zinc in individuals with SD compared to a healthy control group. This research sheds light on the potential roles these nutrients play in the etiopathogenesis of SD, highlighting significant findings, particularly the association of zinc deficiency with SD and the variable influence of vitamin D based on disease severity.

### 4.1 Key Contributions to the Literature

We observed a marked deficiency in zinc among patients with SD, reinforcing its critical role in maintaining skin health, immune function, and microbial balance. Zinc's involvement in protein synthesis, DNA repair, and cellular proliferation is well‐documented, emphasizing its importance in skin integrity and repair. Its anti‐inflammatory and antimicrobial properties, particularly against Malassezia species linked to SD, suggest that zinc deficiency may exacerbate the condition. This aligns with previous reports indicating therapeutic benefits from topical zinc applications, supporting the notion that zinc supplementation could serve both preventive and therapeutic roles in managing SD [13, 14, 15, 16].

In contrast, the relationship between vitamin D levels and SD proved more complex. While no significant difference in overall vitamin D levels was found between SD patients and healthy controls, a stratified analysis revealed lower levels in those with moderate to severe SD. This indicates a potential exacerbating role of vitamin D deficiency, given its involvement in regulating keratinocyte function and immune response in inflammatory skin conditions. The variability in findings across studies, including those by Sobhan et al. [17] and Dimitrova et al. [18], suggests that factors such as receptor sensitivity and genetic variations may influence the relationship between vitamin D and SD. Notably, while Dimitrova et al. (2013) reported low vitamin D levels in patients with exacerbated SD, our study did not find a significant correlation between vitamin D deficiency and disease severity. This discrepancy highlights the variability in findings regarding vitamin D across populations and emphasizes the need for further research to clarify its role in SD [19, 20].

The insufficient levels of 25(OH) vitamin D in patients with SD may be related to isoenzymatic changes associated with the metabolism of vitamin D. Isoenzyme polymorphism has also been shown to affect serum levels of 25(OH) vitamin D. Some studies have demonstrated isoenzyme polymorphism in patients with inflammatory skin diseases such as atopic dermatitis. These findings suggest a potential genetic component influencing vitamin D metabolism in individuals with SD and other inflammatory skin conditions, indicating that genetic variations in enzymes involved in vitamin D metabolism could contribute to the altered vitamin D status observed in these patients. This underscores the complexity of vitamin D metabolism and its interaction with genetic and environmental factors in the context of inflammatory skin diseases [21].

However, no similar study has been conducted for SD. Additionally, studies have not shown any signs of systemic inflammation in patients with SD. Therefore, the deficiency in 25(OH) vitamin D levels is not secondary to inflammation. This indicates that while vitamin D deficiency is observed in patients with SD, it may not be directly linked to systemic inflammatory processes, suggesting that the pathophysiology of vitamin D deficiency in SD could be influenced by factors other than systemic inflammation, such as genetic polymorphisms or alterations in vitamin D metabolism specific to the condition [22].

Rahimi, Nemati, and Shafaei‐Tonekaboni, in their study, demonstrated that serum levels of 25(OH) vitamin D in patients with SD were significantly lower than those in the control group. Furthermore, patients with severe disease had significantly lower levels of 25(OH) vitamin D compared to other patients. This finding underscores the potential role of vitamin D deficiency not just in the occurrence of SD but also in the severity of the condition, suggesting that vitamin D levels could be an important factor in the management and treatment strategies for patients with SD, especially those presenting with severe symptoms [11].

In our study, no significant difference was observed between the serum level of 25(OH) vitamin D and its deficiency among the two groups of patients and controls. However, consistent with the findings of Rahimi, Nemati, and Shafaei‐Tonekaboni [11], it was shown that serum vitamin D levels in patients with moderate and severe forms of the disease were significantly lower compared to those with mild forms. Similarly, Akbaş et al. [3] in their study demonstrated that severe vitamin D deficiency was more prevalent among patients with SD compared to the control group [3]. Although our study found a higher prevalence of severe vitamin D deficiency in patients with SD, the observed difference was not statistically significant. Güder and Güder [23] also showed that there was no significant difference in the serum level of 25(OH) vitamin D and its sufficiency between the groups of patients with SD and healthy controls. The findings of our study are aligned and consistent with those of Güder and Güder, indicating a lack of a statistically significant difference in vitamin D levels and sufficiency between patients with SD and healthy controls.

The study by Karabay and Çerman [5] showed that the serum zinc level in patients with SD is significantly lower compared to the control group, a finding that was also observed in our study. On the other hand, the study by Hajheydari, Saeedi, and Hosseinzadeh [6] did not observe a significant difference in serum zinc levels between patients with SD and the control group, which contrasts with our findings [6]. This discrepancy could be due to differences in the sample sizes, which were larger in our study. Another study by Jahan et al. [7] also did not observe a significant difference in serum zinc levels between patients with SD and the control group, making our findings inconsistent with those of Jahan et al. [7] as well.

Zinc plays a role in various processes that could affect the development of SD. It influences the metabolism of proteins, lipids, and nucleic acids and acts as a cofactor in metalloenzymes and transcription factors. Zinc also plays a role in gene transcription through proteins and factors containing zinc finger motifs, and it regulates cell proliferation, immune activity, and wound healing. We believe that zinc deficiency could play a role in the pathogenesis of the disease through various mechanisms. Furthermore, topical zinc compounds have been reported to be effective in treating SD. Pierard and Pierard‐Franchimont reported that topical zinc formulations might be effective in treating SD through modulation of epithelial differentiation, anti‐inflammatory and antibacterial activity, and inhibition of 5α‐reductase, providing anti‐androgenic activity [24, 25, 26].

The inconsistent findings across studies underscore the complexity of nutrient‐disease interactions in SD and point to the necessity of a more nuanced approach to understanding these relationships. The potential genetic underpinnings, particularly in the context of vitamin D metabolism and response, emerge as a critical area for further investigation. Genetic polymorphisms in vitamin D receptors or in enzymes involved in its metabolism could explain the variability in serum levels and disease impact observed not only in our study but also across the literature. Exploring these genetic factors could lead to a more personalized approach to treating SD, where nutrient supplementation strategies are tailored based on individual genetic profiles [15, 27].

Moreover, our findings raise questions about the potential interplay between zinc and vitamin D in influencing SD pathogenesis. The synergistic effects of these nutrients on immune function and skin health, combined with their independent associations with SD, suggest a complex interaction that could inform novel therapeutic strategies. Future studies should aim to elucidate these interactions, potentially revealing new targets for intervention in SD [28].

In light of our findings, the management of SD may benefit from a holistic approach that considers not only topical and pharmacological treatments but also nutritional interventions. Given the significant impact of zinc deficiency and the nuanced role of vitamin D in SD, assessing and addressing potential deficiencies in these nutrients could enhance treatment outcomes. However, it is essential to approach such interventions with caution, considering the optimal dosages and potential side effects, underscoring the need for further research to establish evidence‐based guidelines [29].

### 4.2 Limitations and Future Directions

Our study has several limitations. The sample size, while adequate, may not capture the full variability of SD across different populations. Additionally, we did not explore the genetic factors that could influence vitamin D metabolism or the specific pathways through which zinc affects SD pathogenesis. Future research should aim to address these gaps, particularly focusing on the genetic underpinnings of vitamin D and zinc metabolism and their interactions in the context of SD. Moreover, our findings raise important questions about the interplay between zinc and vitamin D in influencing SD. The potential synergistic effects of these nutrients on immune function and skin health merit further investigation. Understanding these interactions could inform new therapeutic strategies for managing SD.

## 5 Conclusion

This study provides valuable insights into the relationship between serum levels of 25(OH) vitamin D and zinc in individuals with SD. While no significant difference was observed in vitamin D levels between patients and controls, the notably lower zinc levels in the patient group suggest a potential link between zinc deficiency and the condition. This finding underscores the need for clinicians to consider zinc status when evaluating and managing patients with SD. Given that the severity of the condition correlates with lower vitamin D levels, further research is warranted to explore the role of vitamin D supplementation in improving disease outcomes. Future studies should aim to investigate the effectiveness of zinc supplementation as a therapeutic intervention, particularly for patients exhibiting zinc deficiency. Additionally, larger sample sizes are needed to confirm these trends and to better understand the multifaceted role of these nutrients in skin health. Overall, these findings could influence clinical guidelines by advocating for routine screening of zinc levels in patients with SD and considering nutritional interventions as part of a comprehensive management strategy. Such steps may enhance patient care and provide a foundation for future research into the nutritional aspects of dermatological conditions.

## Author Contributions

**Amine Kashiri:** conceptualization, methodology, data collection, writing–original draft. **Negin Maghsoudloo:** data analysis, review and editing.

## Conflicts of Interest

The authors declare no conflicts of interest.

### 1 Transparency Statement

Amine Kashiri affirms that this manuscript is an honest, accurate, and transparent account of the study being reported. No important aspects of the study have been omitted, and any discrepancies from the study as planned (and, if relevant, registered) have been fully explained.

## Data Availability

The data that support the findings of this study are available from the corresponding author upon reasonable request. All authors have read and approved the final version of the manuscript. The corresponding author, Amine Kashiri, had full access to all of the data in this study and takes complete responsibility for the integrity of the data and the accuracy of the data analysis.
